# Pilot of rapid implementation of the advanced practice provider in the workflow of an existing tele-critical care program

**DOI:** 10.1186/s12913-022-08251-4

**Published:** 2022-07-02

**Authors:** Krzysztof Laudanski, Ann Marie Huffenberger, Michael J. Scott, Justin Wain, Danyal Ghani, C. William Hanson

**Affiliations:** 1 Department of Anesthesiology and Critical Care, University of Pennsylvania, Philadelphia, PA 19104 USA; 2 Leonard Davis Institute for Health Economics, Philadelphia, PA 19104 USA; 3 Department of Anesthesiology and Critical Care, Leonard Davis Institute for Health Economic, JMB 127; 3620 Hamilton Walk, Philadelphia, PA 19146 USA; 4 School of Osteopathic Medicine, Campbell University, Buies Creek, NC 27506 USA; 5 Penn Medicine Center for Connected Care, Hospital of the University of Pennsylvania, Philadelphia, PA 19104 USA; 6 College of Art & Sciences, Drexel University, Philadelphia, PA 19104 USA

**Keywords:** Tele-critical care medicine, ICU, Advance practice providers, Workflow, Operations, Implementation

## Abstract

Incorporating the advanced practice provider (APP) in the delivery of tele critical care medicine (teleCCM) addresses the critical care provider shortage. However, the current literature lacks details of potential workflows, deployment difficulties and implementation outcomes while suggesting that expanding teleCCM service may be difficult. Here, we demonstrate the implementation of a telemedicine APP (eAPP) pilot service within an existing teleCCM program with the objective of determining the feasibility and ease of deployment. The goal is to augment an existing tele-ICU system with a balanced APP service to assess the feasibility and potential impact on the ICU performance in several hospitals affiliated within a large academic center. A REDCap survey was used to assess eAPP workflows, expediency of interventions, duration of tasks, and types of assignments within different service locations. Between 02/01/2021 and 08/31/2021, 204 interventions (across 133 12-h shift) were recorded by eAPP (n_routine_ = 109 (53.4%); n_urgent_ = 82 (40.2%); n_emergent_ = 13 (6.4%). The average task duration was 10.9 ± 6.22 min, but there was a significant difference based on the expediency of the task (F [2; 202] = 3.89; *p* < 0.022) and type of tasks (F [7; 220] = 6.69; *p* < 0.001). Furthermore, the eAPP task type and expediency varied depending upon the unit engaged and timeframe since implementation. The eAPP interventions were effectively communicated with bedside staff with only 0.5% of suggestions rejected. Only in 2% cases did the eAPP report distress. In summary, the eAPP can be rapidly deployed in existing teleCCM settings, providing adaptable and valuable care that addresses the specific needs of different ICUs while simultaneously enhancing the delivery of ICU care. Further studies are needed to quantify the input more robustly.

**Supplementary Information:**

The online version contains supplementary material available at 10.1186/s12913-022-08251-4.

## Introduction

As the United States faces increased physician shortages, one successful solution has been to allocate patient care to nurse practitioners (NPs) and physician assistants (PAs), otherwise known as advanced practice providers (APP) [1]. APPs have enjoyed an increasingly sizable percentage of the healthcare workforce in the United States. Similar trends are seen in other countries, where increasing number of APPs are seen providing healthcare services [1–3]. Depending upon the state and hospital regulations, APPs perform a variety of healthcare services, ranging from evaluating and diagnosing to developing treatment plans [4–7]. APPs have demonstrated the ability to deliver effective and valuable care in various clinical situations, allowing their role to evolve extensively [8, 9]. However, their practice is often complicated by credentialing, licensing, and reimbursement barriers [5, 10]. However, even after their increasing integrations and post-COVID-19 innovation acceleration in healthcare system, patients often have trouble in accessing services [11–13]. These shortages can be addressed by APP depending on the state and hospital regulations [6, 8, 14]. Kreeftenberg et al. [1], Landsperge et al. [9], Mileski et al. [14] and Gershengorn et al. [15] Primary reasons for any limitations include that the practice is often complicated by credentialing, licensing, and reimbursement barriers [16]. However, even if these barriers are reduced, the demand for healthcare service outstrips available healthcare delivery by various providers [11, 13]. Therefore, another solution is needed to multiply the capacity of the existing workforce while continuing to deliver a high quality of care.

The healthcare field has strategically expanded the role of APPs into the critical care setting to meet the increasing demands for ICU provider resources across the United States [1, 15]. Given the success of APPs at the bedside, it is interesting to find a relatively slow transition of APPs into a remote or teleCCM setting [9, 17]. The COVID-19 pandemic provided impetus to incorporate APPs into the structure of telemedicine considering the physician shortage, the need to transfer patient care to a remote setting, the creation of non-ICU locations, and implement strategies to prevent long-term staff fatigue and shortages [3, 16, 18]. Furthermore, a survey conducted by the American Association of Nurse Practitioners between July 28, 2020 and August 9, 2020, showed that 63% of approximately 4,000 nurse practitioners who responded indicated that they continued to transit patients to telemedicine rather than in-person visits. Although most APP work is in primary care, their expertise could be applied to the tele-Critical Care Medicine (teleCCM) system [18–21]. However, the relative paucity of data presenting integration of APP services into tele-ICU programs may be secondary to the insufficient record of publications as several programs that currently employ APPs in their teleCCM structure have failed to publish a peer-reviewed record of such an endeavor [17]. Incorporating APPs into teleCCM delivery could address several of the supply and demand problems associated with ICU providers, but data is lacking on potential workflows and outcomes, especially about the incorporation of their services in the existing model that would augment the teleCCM system [18].

Telemedicine is a modality of healthcare delivery that provides healthcare services to patients from a remote location, using audio–video technologies, although this definition may vary by organization [3]. Telemedicine has become an increasingly adopted strategy among hospitals due to its cost-effectiveness, adaptability, and efficiency [3, 18, 22–25]. These features require substantial initial costs to deploy the infrastructure. However, once deployed, the tele-ICU infrastructure can be utilized in several ways at significantly less expense [26, 27]. It is an evolutionary step in virtually all tele-ICU programs to seek additional ways to utilize and expand the service using non-physician providers [15, 20]. Doing so reduces the total cost of care. Furthermore, the increase in effectiveness of teleCCM services by employing a variety of providers was critical to ensuring the success of tele-ICU during the pandemic [18, 26].

One of the suggested approaches to developing tele-ICU is exploring the incorporation of various healthcare providers [18]. Interestingly, pharmacists, respiratory therapists, and data coordinators are all part of the tele-ICU team, which demonstrates the flexibility of tele-ICU to incorporate various providers [18, 27]. Considering the significant and growing role of APPs in U.S. healthcare, their entry into tele-ICU is expected. However, there is a knowledge gap about the performance and potential workflows of telemedicine APP (eAPP) in ICU settings [18, 20, 22, 24]. Knowledge and implementation experience are often siloed within the organizations and their associated healthcare systems, with little dissemination to others to learn the processes for implementing this type of care. It is also unknown whether the rapid implementation of teleCCM providers can be done quickly on a need-to-provide services basis. Finally, there is gap in knowledge about whether the eAPP can adapt over time to the specific needs and culture of the ICU to best address patient’s needs in the teleCCM setting [28].

Here, we describe a pilot project of an *ad hoc* implementation of APP into the structure of an existing teleCCM program (Penn e-Lert), using a predetermined set of interventions or tasks tailored to the need of the existing healthcare system as well as ongoing patient needs. Thus, we challenged the idea that the deployment of teleCCM services is an arduous and complex task. Finally, we hypothesized that their scope of engagement will evolve over time to adapt to the specific needs of ICUs [18, 28].

## Materials and methods

### Description of telemedicine setting

The Penn e-Lert tele critical care medicine team (teleCCM) at the University of Pennsylvania Health System (UPHS) utilizes a hybrid care model aimed to provide reactive (33%), proactive (33%), and quality assurance approaches (33%) [27–29]. The bedside care providers retain full responsibility for the direct care of the patients while the teleCCM staff provides consultative services. An already-in-place teleCCM Penn e-Lert consists of medical doctors (eMD), registered nurses (eRN), and respiratory therapists (eRT) with a critical care background.

### eAPP workflow

For this pilot study, Penn e-Lert employed bedside APPs in a moonlighting model to help support ICU bedside staff during a high census (02/1/2020 and 8/31/2021) with the utilization of teleCCM technology. The eAPP staffing model aimed at providing 7:00PM to 7:00AM coverage seven days a week. A total of 133 of 12-h shifts were included in this report.

eAPP remote interventions were triggered by input from the bedside staff (considered a reactive response), the autonomous algorithm for ARDS detection, utilizing predetermined clinical queries (considered a proactive response), or actively reviewing patients' data. The latter task is focused on ensuring practice compliance, but it can trigger a proactive response based on the eAPP’s assessment (Supplemental Fig. 1). The eAPP utilized a tele-medicine system, electronic medical records, and remote audio-visual surveillance to assess clinical status, medications, available imaging, laboratory measurements, as well as current and prior medical notes as needed. An audio–video system allows for the real-time assessment of a patient and provides real-time communication with bedside staff, patients, and families (Supplemental Fig. 1).

eAPP action can be triggered by the bedside staff (via an in-room push button, a phone call, the asynchronous texting platform, or the teleCCM system, providing an alert based on vital signs and lab values—an algorithmic sniffer for the detection of ARDS. The expediency of the intervention was deemed routine (completed within 2 h), urgent (to be completed within 15 min), and emergent (to be addressed without delay) (Supplemental Fig. 1).

Tasks would be classified accordingly into existing workflows including pre-existing protocol (Targeted Temperature Management, Acute Respiratory Distress Syndrome/low stretch ventilation, cardiac arrest), helping with patient management (shock support, unstable), admission to the hospital, generally defined support/oversight, otherwise not classified (Supplemental Fig. 1).

If the eAPP deems it necessary to communicate with the bedside staff, several modalities exist, including audio–video technology, mobile phones, asynchronous messaging, or relay messages via electronic medical records. The eAPP was able to communicate with both bedside staff and Penn e-Lert professionals (Supplemental Fig. 1). Interventions or tasks could be classified into two of six categories: *a clinical intervention, referring* to any action affecting patient care delivery; *quality and assurance,* denoting assurance of best practice compliance; *safety* assigned to tasks that require intervention in addressing potential harm; *education* capturing any eAPP-led teaching; *debriefing* when bedside staff wish to review particularly difficult clinical cases; and *recording* for documenting clinical situations. O*ther* categories are used to capture all else (Supplemental Fig. 1).

### eAPP implementation

The staff was briefed about the operation of the Penn e-Lert during their initial shift. They were encouraged to start virtual rounds and respond to clinical cues from teleCCM system. The workflow was modelled by the existing culture of the Penn e-Lert, which is a mix of proactive, reactive, and quality assurance tasks. The eAPP encouraged actively looking for engagement opportunities to prevent patient clinical deterioration, assist with care, and assure quality and harm prevention in accordance with the UPHS philosophy of being a high reliability organization.

### Data collection

Once inventions or tasks were completed, eAPP entered the characterization of the conducted intervention in the REDCap database, using a structured format [30]. The duration of the task, its complexity, and the perception of how the recommendation was utilized during engagements (accepted, acknowledged, or rejected) along with the level of eAPP distress were recorded (Supplemental Material 1). The eAPP survey was adopted from the Penn e-Lert survey used to monitor the workflow of other healthcare professionals. The entries should be completed after each intervention, yet compliance with this task was not checked. The data was collected between 02/1/2020 and 8/31/2021.

### Statistical analysis

Descriptive analysis was used throughout. The Shapiro–Wilk W test and distribution plots tested the normality of distribution variables. Parametric variables were expressed as mean ± SD. Median (Me) and interquartile ranges (I.R.). In some cases, ANOVA was utilized to compare means. Frequencies were compared, using χ^2^ if the incidence of an occurrence was at 5 at minimum. Statistical analyses were performed with Statistica 11.0 (StatSoft Inc., Tulsa, OK).

## Results

### Analysis of the eAPP involvement into care delivery

**Fig. 1:**
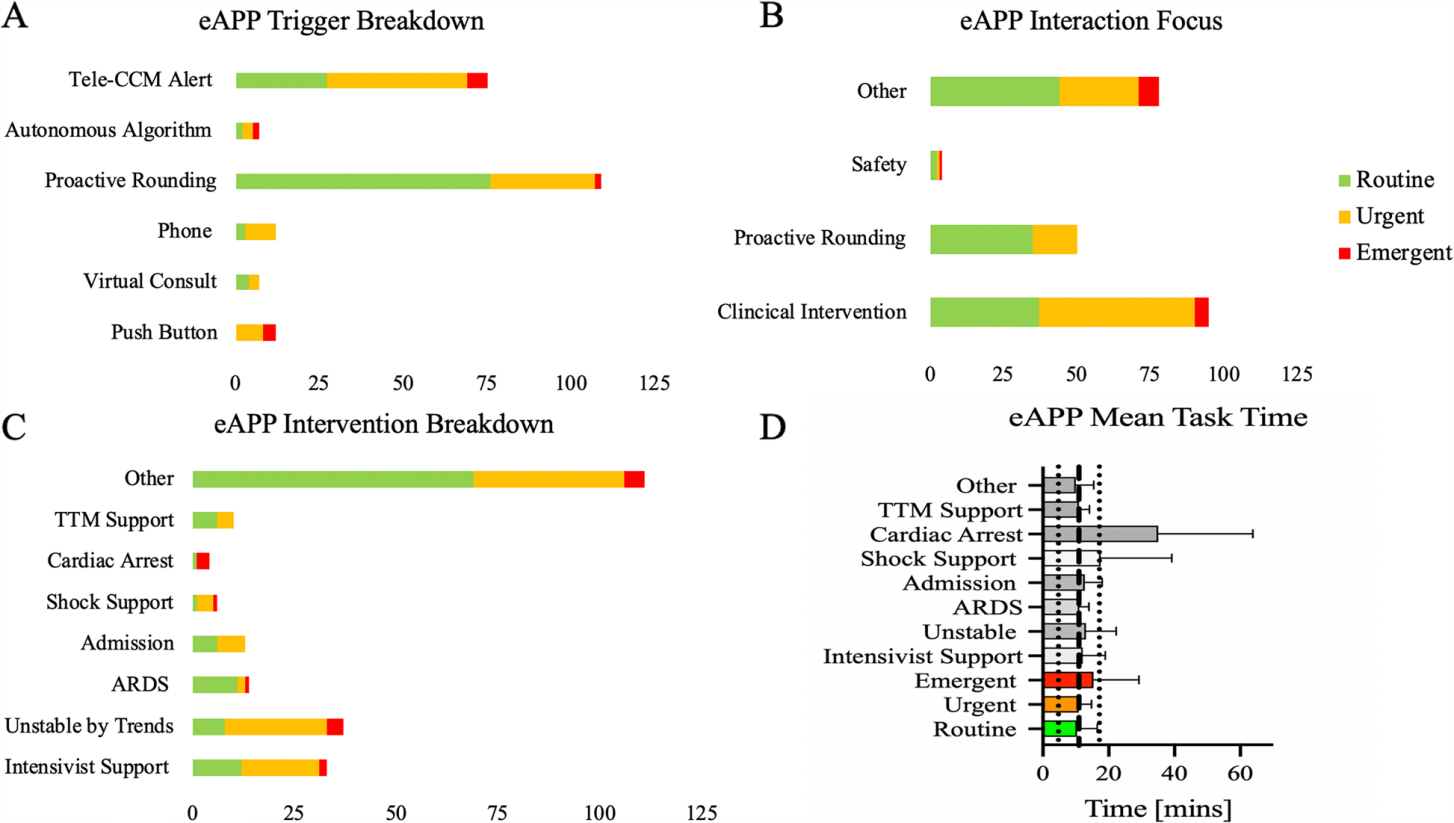
eAPP interventions were triggered in several ways (**A**) and were a mix of other, pro-active rounding and clinical interventions (**B**). Specific tasks were mostly unspecified but with a significant number unstable by trends and intensivist support (**C**). The time on the task was significantly different when cardiac arrest was considered, while other tasks had a similar duration (**D**)

Between 02/01/2021 and 08/31/2021, 204 interventions were recorded by the eAPP with a variable level of expediency (n_routine_ = 109 (53.4%); n_urgent_ = 82 (40.2%); n_emergent_ = 13 (6.4%). Most tasks were triggered by proactive rounding (48.9%), followed by the teleCCM platform generating best practice alerts (33.6%) (Fig. 1A). For interventions deemed urgent or emergent, the eAPP was notified by a Penn e-Lert staffing triage or by push buttons at 100% of the time (Fig. 1A). Clinical interventions were the primary focus in 41.7% of eAPP engagements, while 34.2% of eAPP interventions involved a clinical focus not otherwise listed (Fig. 1B). When clinical intervention was the focus of the engagement, 61% of the time it was considered urgent or emergent (Fig. 1B).

As for the eAPP, proactive rounding was the primary focus at only 21.9%. In terms of task type, the vast majority of tasks were categorized as *others* (Fig. 1C). For the tasks described as *support*, *unstable patients*, or *shock support*, they were deemed urgent or emergent 63.6%, 78.4% and 83.3% of the time, respectively (Fig. 1C). Cardiac arrest had an expectantly high number of emergent entries (Fig. 1C).

On average, the eAPP spent 10.9 ± 6.22 min per task, but there was a statically significant difference in the time spent per task depending upon its expediency (F [2; 202] = 3.89; *p* < 0.022) and type (F [7; 220] = 6.69; *p* < 0.001) (Fig. 1D).

### Means and acceptance of communication between eAPP and bedside staff

In 5.2% of eAPP engagements, no follow-up communication took place. When communication did take place, it occurred with the RN or eRNs 27.3% of the time, followed by bedside staff attending or eMDs at 21.0% of the time, Next, with the APP, CRNA, or eAPP, it was 21.0% of the time; and last, with the RT or eRT, it reached 14.8% of the time, withy 63% over the telephone.

The perception of the eAPP is that the recommendations were straightforwardly accepted at 33.9% of the time. In 27.2% of cases, they acknowledged that the eAPP could not determine the impact at 38.4%. Rejecting an intervention occurred at 0.5% of the time. In 2% of cases, the eAPP noted a distress related to management.

### Adaptation of the eAPP workflow to ICU characteristic over time

**Fig. 2:**
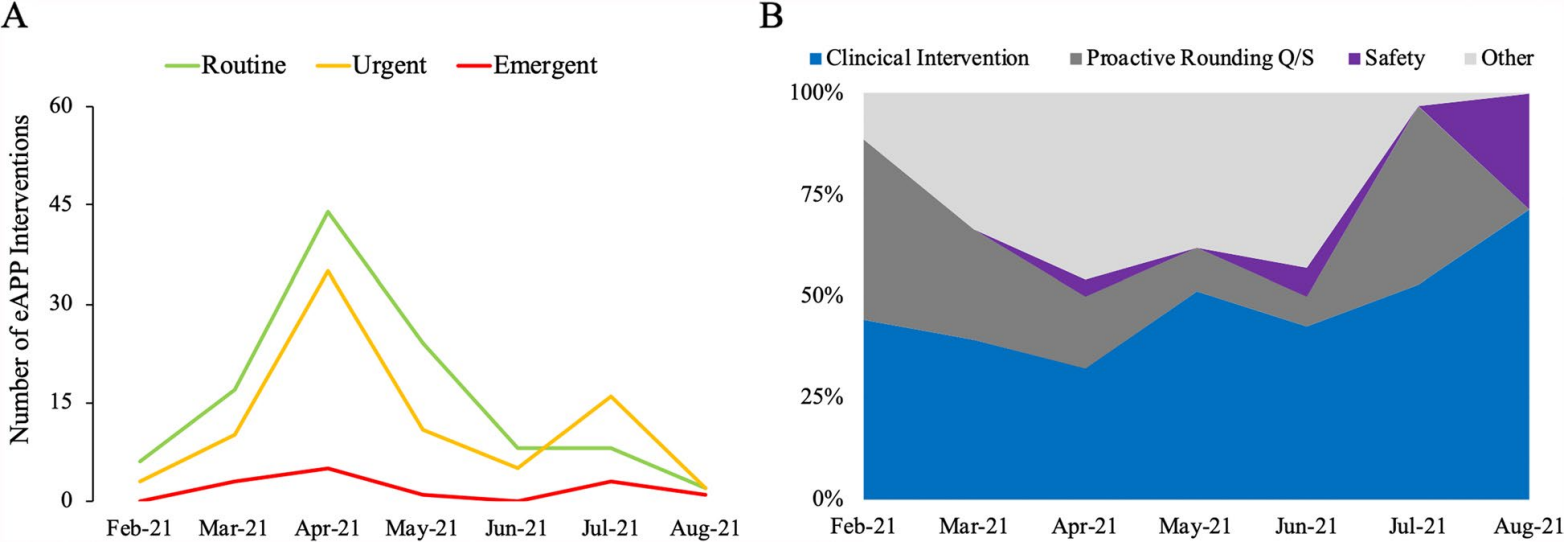
During duration of the pilot study an increase in routine and urgent cases in April was seen (**A**) while proactive rounding became more common as an eAPP focused at the beginning and end of the pilot (**B**)

The eAPP saw an increase in routine and urgent cases in the month of April, while emergent cases remained relatively the same throughout the remaining pilot period (Fig. 2A). Also, the composition of the tasks changed over the pilot study (Fig. 2B).

**Fig. 3:**
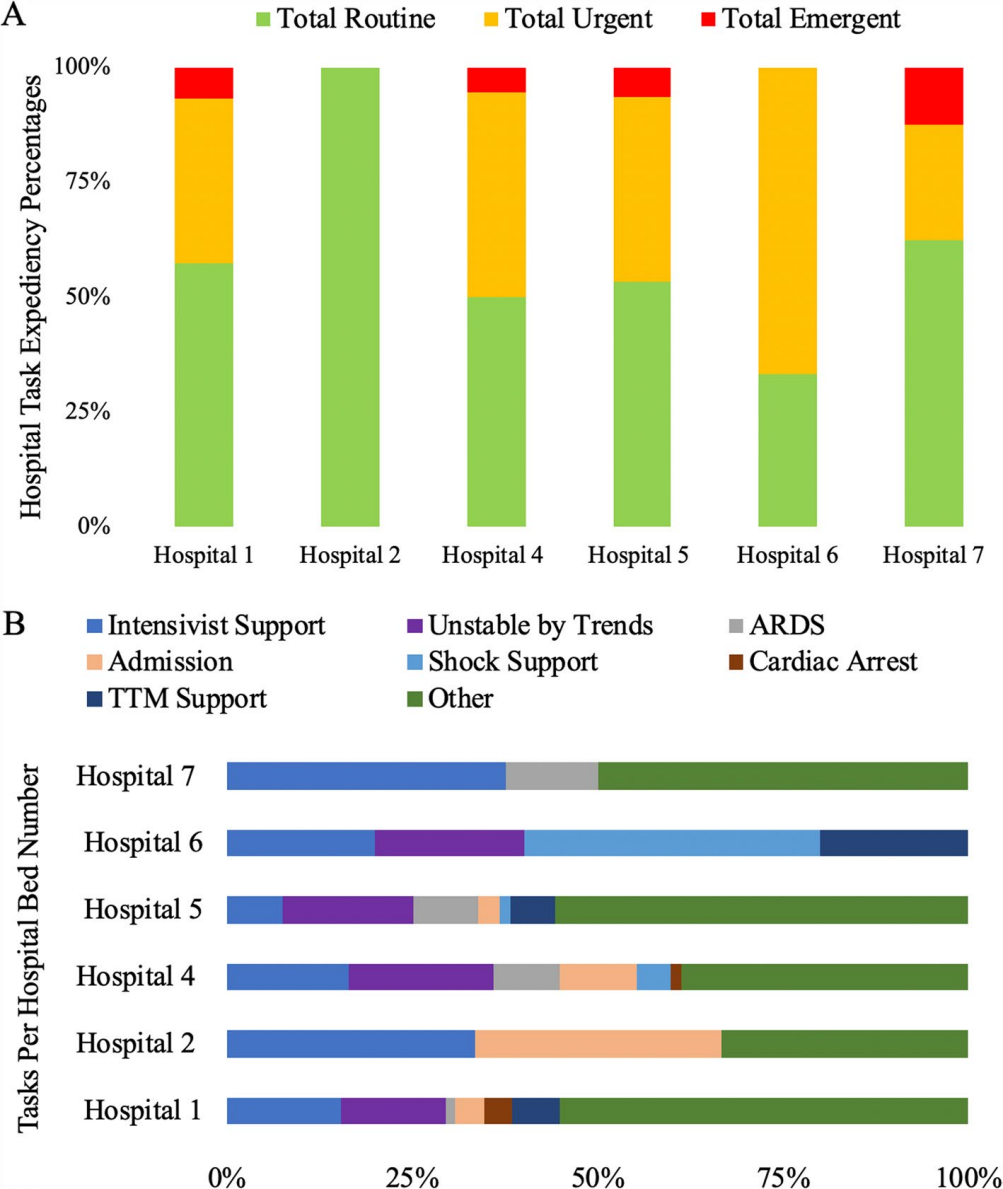
Expediency of engagements varied across the different hospital locations (**A**). Other tasks not listed were the most utilized across the majority of hospitals, while hospital 6 showed a higher incidence of tasks, focusing on shock support (**B**)

When analyzing eAPP service across different hospital locations during the pilot, we saw that the expediency of interventions and task type varied although not in a signfiicantly statistical way. It was noted that 66.7% of the eAPP engagements at Hospital 6 were urgent in nature (Fig. 3A) with shock support being the major task focus 40% of the time (Fig. 3B). Besides Hospital 6, the eAPP tasks otherwise not listed made up the majority of those completed (Fig. 3B). Hospital 7 saw the greatest number of tasks requiring intensivist and ARDS support. Hospital 2 saw similar intensivist support tasks as Hospital 7, but Hospital 2 saw the greatest number of admissions compared to the other locations (Fig. 3B). Although there is a total of 7 hospitals, Hospital 3 did not report any engagements requiring the eAPP involvement.

**Fig. 4:**
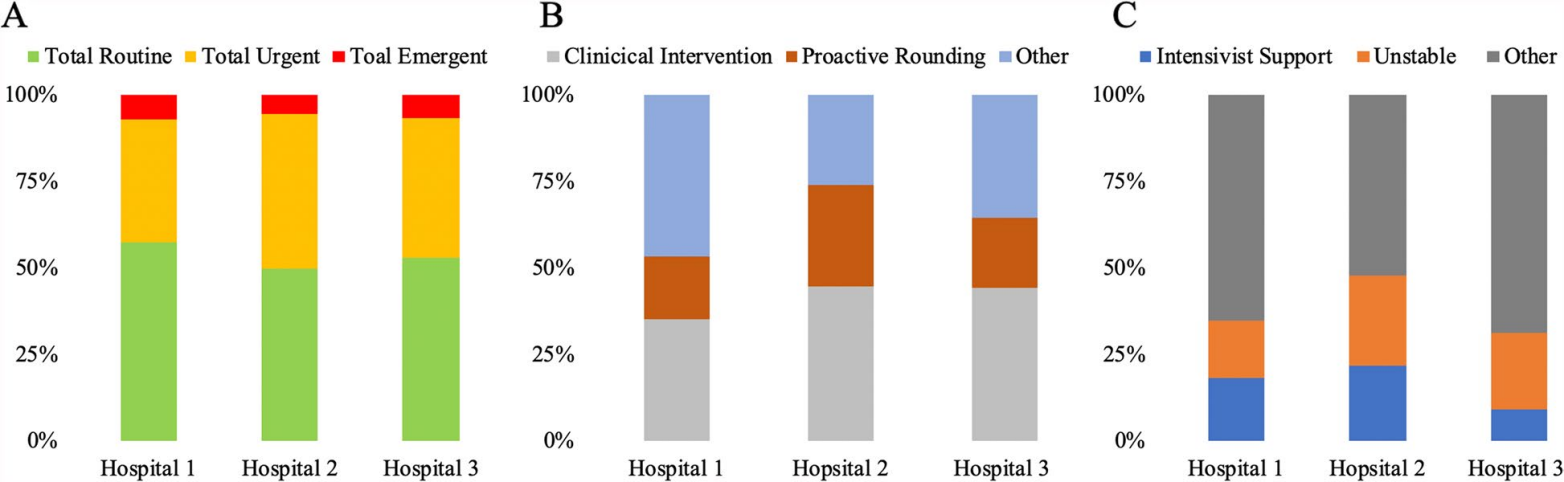
Expediency and type of engagements varied insignificantly across the different hospitals (**A**&**B**), similarly to type of intervention (**C**)

When reviewing eAPP’s responses across the three largest hospitals, we found that eAPPs had similar frequency of routine, urgent, and emergent across all three systems (χ2 [4;191] = 1.114; *p* = 0.89) (Fig. 4A).

The tasks focused on clinical intervetnion were similar across all three hospital systems as no trends reached the level of statistical significance. Hospital 2 saw more tasks focused on proactive rounding, while Hospital 1 had more tasks deemed “other;” but these differences were not statistically significant (Fig. 4B). Hospital 1 also had more interventions deemed “other”, similar to Hospital 3, while Hospital 2 had more interventions dealing with unstable patients (Fig. 4C).

### eAPP involvement in the care of the patient during COVID-19 pandemic

**Fig. 5:**
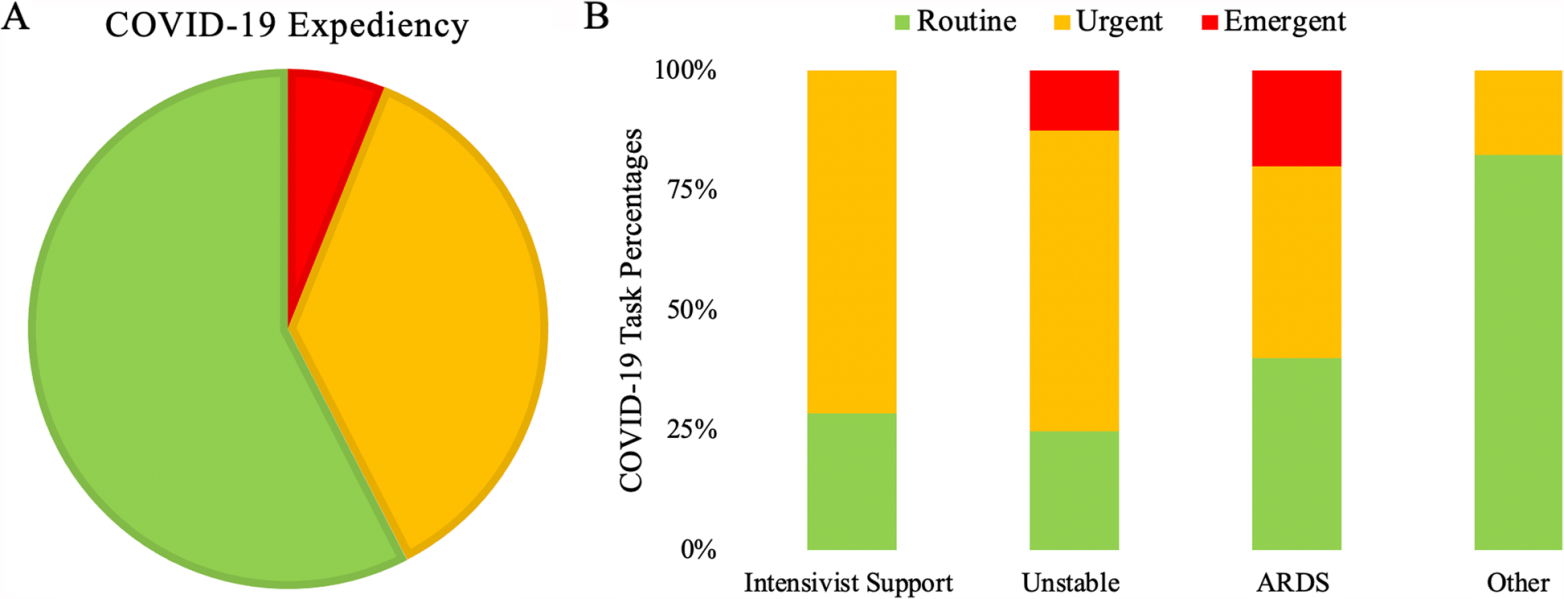
eAPP expediency breakdown for COVID-19 positive patients (**A**). eAPP tasks for COVID-19 patients varied if they were considered routine, urgent, or emergent (**B**)

The eAPP engagements involved COVID-19 patients 16.2% of the time; 42.4% of the time these engagements were labeled urgent or emergent (Fig. 5A). The majority of tasks associated with COVID-19 patients were listed as other 45.9% of the time. In engagements deemed urgent or emergent, 35.5% of the time, the focus of the main task was on unstable patient trends, 29.4% on intensivist support, and 17.6% on ARDS (Fig. 5B).

## Discussion

To our knowledge, this is the first report that describes the performance of the eAPP in teleCCM settings, utilizing *ad hoc* deployment and the hospital system demand-driven process. Prior reports assessed the eAPP for a large system with a pre-determined deployment [17]. However, such a model requires significant upfront expenditures and system investments that represent a barrier for incorporating an eAPP in patient care [16, 21, 26]. This is in stark contrast to the presumption of telemedicine as being easily deployed, expandable, and adaptable [18, 26]. Our study demonstrated that the APP can be included in the teleCCM workflow and provide a valuable service. In fact, it can be deployed rapidly to address the needs of the several hospitals and ICUs.

We utilized the existing structure of the Penn e-Lert in terms of hardware, software, operating principles, and the means to assess care delivery. In our assessment, the integration of the eAPP was seamless and able to deliver value to patients. At the conclusion of the pilot, the healthcare system decided to convert the eAPP from *ad hoc* deployment to a permanent structure as part of building a “high reliability organization”. To our knowledge, this is the first-time an intervention was assessed despite multiple suggestions to establish the timeframe of teleCCM providers [18]. While the assessment was subjective, it does establish a reference, which may be useful in planning future eAPP deployment and expectations in respect to how many cases one provider can manage.

Most eAPP engagements were related to routine and urgent tasks, reflecting assigned work, consistent with the initial service intent. The average engagement was around 10 min, but significant differences existed depending upon task expediency and type. This is expected as emergent tasks need to be addressed for a longer period given that they are more complex in validating data. Most of the interventions related to shock were described as emergent and urgent. This is expected, considering the very dynamic nature of these health states and like the bedside experiences by APP [1, 2, 17]. The number of routine tasks dominated slightly, but this was expected, considering that the eAPP workflow was a hybrid of the routine rounding and Penn e-Lert workflow [28]. This is the first time that data pertaining to eAPP workflow and engagement parameters has been published.

The eAPP was able to interact effectively with all stakeholders of the bedside team via several means. This is a key component of successful deployment [31]. The level of distressful interaction was around 2%, while only 0.5% of recommendations were rejected. However, 34% of intervention recommendations were accepted while 27.2% were merely acknowledged and 30.6% of interventions were not classified by the eAPP. The level of acceptance was lower than expected but then we used a subjective assessment. We believe that increasing the brief and outreach is important to increasing acceptance as bedside staff frequently reported a lack of awareness about the eAPP during post-hoc interviews [21, 31]. This underscores the need for a pre-deployment briefing for all providers with clearly established goals, expectations, and roles before and after establishing the eAPP [3, 21, 28].

It is important that the distress level was infrequent among the eAPP. This is particularly important as a high number of engagements in a fully developed teleCCM practice may result in burnout among the providers [12]. Our study gives first-time metrics of acceptance and distress rates for the eAPP in a teleCCM setting, but it also demonstrates the need for the deployment of a more robust tool to assess eAPP effectiveness and burnout in the next study as we had to rely on eAPP perceptions. In the next deployment implementation of performance measures tied to best practice is planned as well to address the clinical impact of the interactions [14, 23].

Over time, the eAPP exhibited increased flexibility as the percentage of proactive rounding increased, which provided the clinical opportunity for other tasks to be completed concurrently. This is similar to bedside observation [1, 16]. Though eAPP was intended to balance the workload during the COVID-19 wave, we saw them often participating in the care of non-COVID-19 patients. This reflects the increased adaptability of the service as the eAPP proactively provided the healthcare service and adapted to the needs in Penn e-Lert semi-structured settings [1]. This is consistent with the culture and mission of Penn e-Lert where remote providers are encouraged to look for novel ways to provide the healthcare service on top of the predetermined tasks assigned [28]. Also, the eAPP may be an ultimately most adaptable medical professionals considering their broad scope of practice [2, 9, 15, 17, 18]. Our study suggests that the eAPP can adapt to the ICU culture and need of different ICU and hospital as demonstrated by their tasks profile changing over time. Our study was not intended to examine the sources of these inter-hospital and inter-ICU differences.

However, it is more than likely that operational flow, culture, leadership, professional staff composition, patient mix, and protocols are different between them resulting in dissimilar needs [19, 32, 33]. Pro-active and re-active care delivery seen in our study is an example of adaptability as the eAPP could engage in pre-determined care and respond to the specific needs of the ICU. This is critical as the regionalization of services is apparent in our system. However, it is unclear how our experiences can be transferred to other system considering that Penn e-Lert has culture of delegating to staff the best way to utilize the teleCCM in order to find and address gaps in care delivery [28].

Our study also has limitations. During this pilot, the eAPP service was not consistent, and due to the demand for APP resources within the health system, many eAPP shifts went unfilled. Because of the uncertain nature of staffing with moonlighting providers, the Penn e-Lert team could not depend on having eAPP service each night. We were also unable to gather qualitative data to present the perceptions of other hospital employees as to the benefits and impacts eAPPs has had on the healthcare team. This study also relied on the REDCap survey being completed by eAPP, which may introduce bias [34–36]. We attempted to minimize it in several ways. All providers received the same introduction and onboarding process to the Penn e-Lert system. Following this onboarding, the interpretation of the REDCap survey was provider dependent, creating an observer bias.

While recall bias should be minimal due to eAPP’s completing the survey shortly following an interaction, selection bias could be significant depending upon whether the eAPP decided to complete the survey following the intervention. Unfortunately, there was no way to verify their activities because the eAPP was not required to indicate completion the survey in their charts [36]. In our discussion with the eAPP team, it became clear that the survey was not done all the time, with a bias was towards attending to capture “more severe or important” issues. Considering that we estimate that 50% of entries might be missing, it may result in significant bias. The missing entry estimate is done by comparing eAPP frequency to other team members like the eMD or eRN.

One of the potential reasons for poor completion of the survey tool was the perception that the eAPP assignment within Penn e-Lert operations was only temporary. Thus, we were challenged to define and operationalize consistent eAPP workflows [28]. Consequently, several entries were characterized as “other”. We hoped that an analyzation of tasks indicated as “other” would show ways that the eAPP works outside characterized tasks and shed light on potential assignments to be further addressed by the eAPP in the teleCCM setting. What we found were recorded comments for “other” tasks that described the situation rather than the task process. These comments were very heterogenous in nature, situational dependent, and not able to provide analyzable data.

Finally, we did not quantify the clinical impact of the eAPP [1, 23]. However, the frequent engagement in emergent and critical situations in response to the emergency button pushed by bedside staff suggests that they were essential in several critical situations. Further studies should aim to provide more quantifiable results in terms of the clinical impact performance of the eAPP. In addition, we did not quantify the effect of the model’s financial basis. Deploying the eAPP should be more competitive than the bedside APP service for this model to be viable. Though teleCCM allows for enhancement and a multiplier effect for healthcare providers, this must be determined in the next study iteration.

## Conclusions

The eAPP can be rapidly deployed in existing teleCCM settings, providing an adaptable and valuable care model that can address the specific needs of different ICU populations.

## Supplementary Information

**Additional file 1: Supplemental Figure 1.** The example of the workflow in Penn e-Lert was re-purposed for eAPP deployment.

**Additional file 2: Supplemental Material 1.** The REDCap tool was utilized to collect info about eAPP activities in the context of their workflow.

## Data Availability

The datasets used and/or analyzed during the current study are not publicly available due containing operational data of the University of Pennsylvania Hospital, but it can be made available from the corresponding authors upon reasonable request and the specific party signing an NDA agreement.
