# Supplementary figures and images for: Pilot of rapid implementation of the advanced practice provider in the workflow of an existing tele-critical care program
Source: BMC Health Serv Res. 2022 Jul 2;22:855. doi: 10.1186/s12913-022-08251-4 (PMC9250728; doi:10.1186/s12913-022-08251-4)

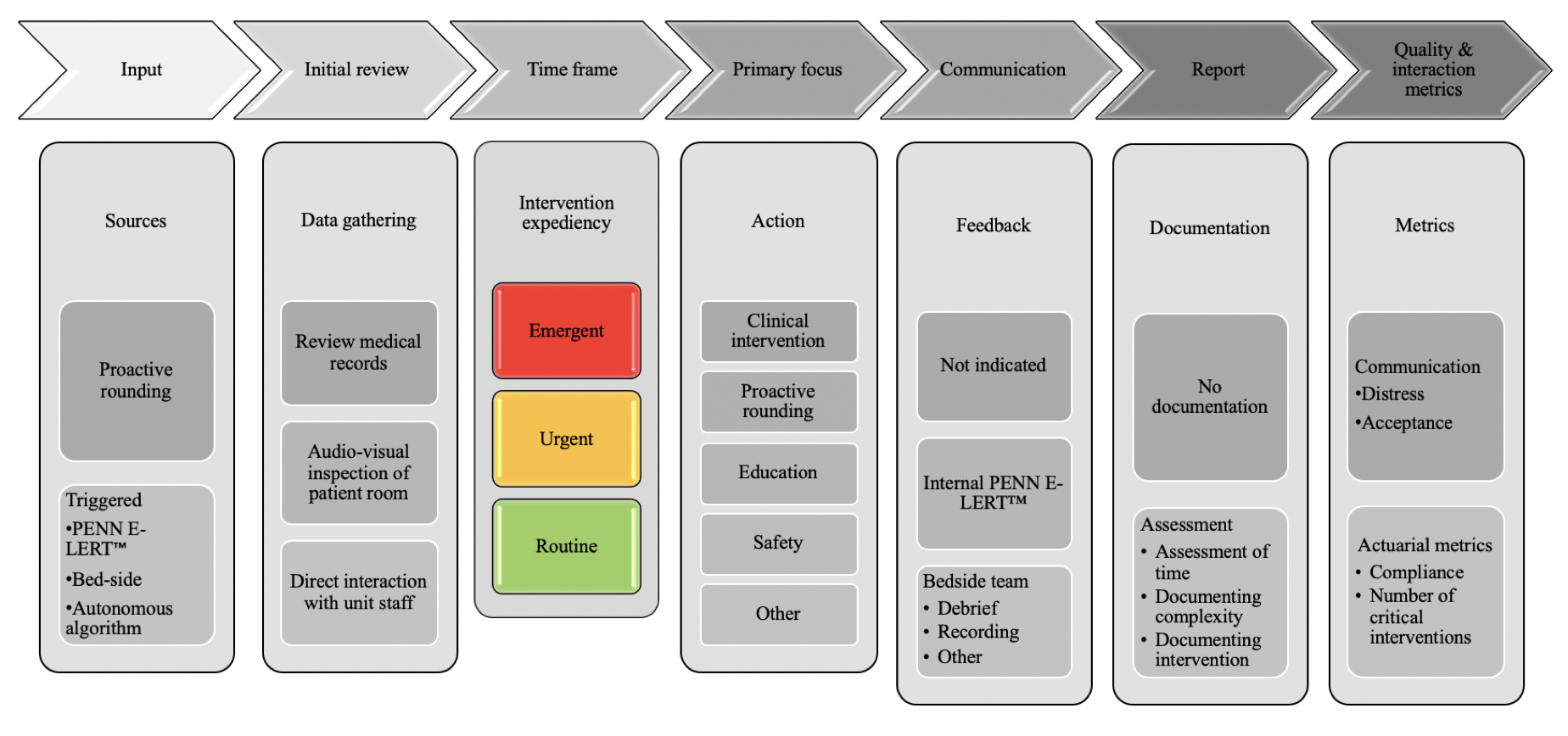

Supplement: Supplementary file 1 — Additional file 1: Supplemental Figure 1. The example of the workflow in Penn e-Lert was re-purposed for eAPP deployment. [file 12913_2022_8251_MOESM1_ESM.png]

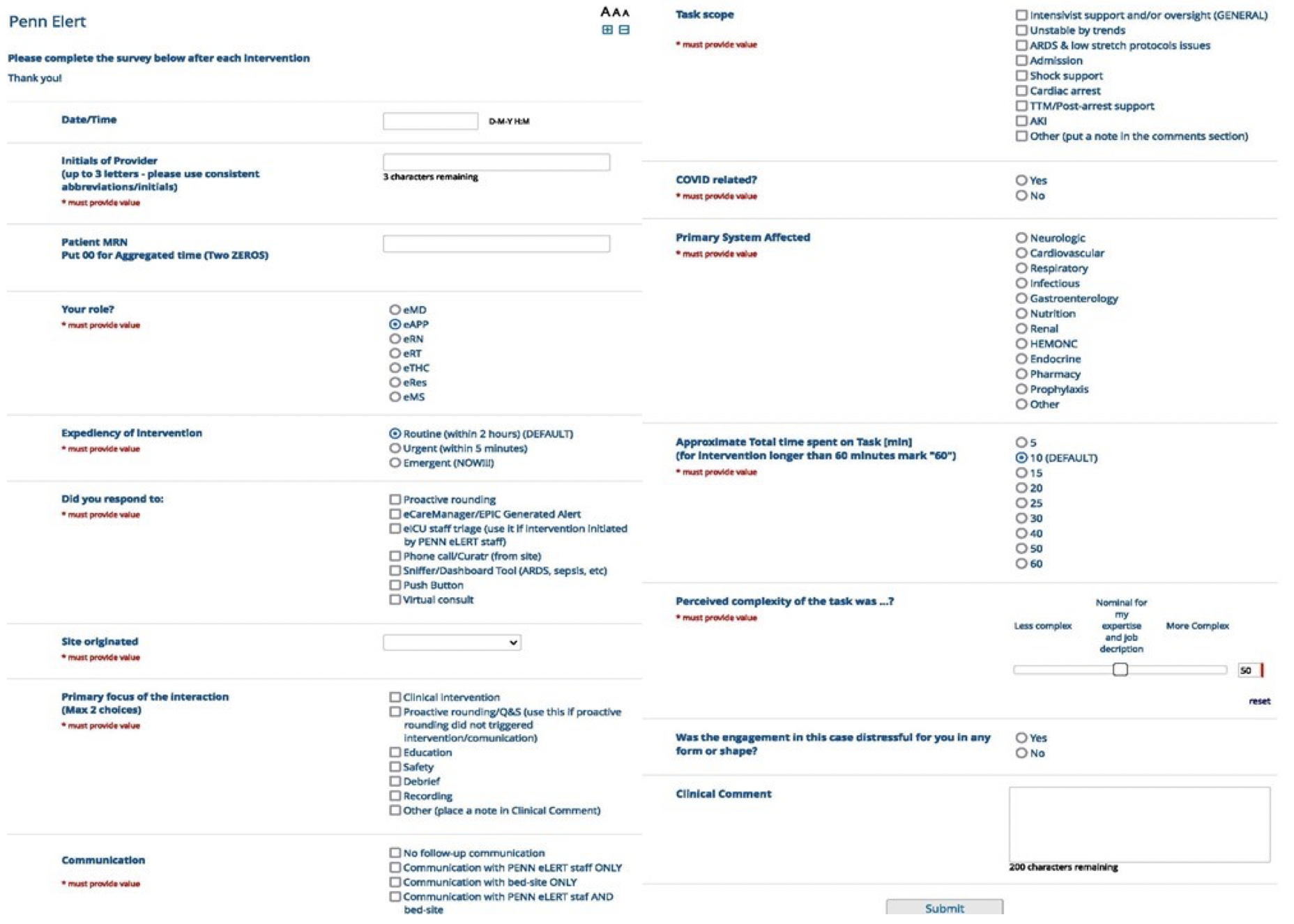

Supplement: Supplementary file 2 — Additional file 2: Supplemental Material 1. The REDCap tool was utilized to collect info about eAPP activities in the context of their workflow. [file 12913_2022_8251_MOESM2_ESM.png]
